# Supplementary material for: Unsplit superconducting and time reversal symmetry breaking transitions in Sr2RuO4 under hydrostatic pressure and disorder
Source: Nat Commun. 2021 Jun 24;12:3920. doi: 10.1038/s41467-021-24176-8 (PMC8225887; doi:10.1038/s41467-021-24176-8)
Supplement: Supplementary file 1 — Supplementary Information [file 41467_2021_24176_MOESM1_ESM.pdf]

Supplementary Information for: "Unsplit superconducting and time reversal symmetry breaking transitions in Sr_2RuO_4 under hydrostatic pressure and disorder."

Vadim Grinenko,[1,2,*] Debarchan Das,[3] Ritu Gupta,[3] Bastian Zinkl,[4] Naoki Kikugawa,[5] Yoshiteru Maeno,[6] Clifford W. Hicks,[7,8] Hans-Henning Klauss,[1] Manfred Sigrist,[4,†] and Rustem Khasanov[3,‡]

[1]*Institute for Solid State and Materials Physics, Technische Universität Dresden, D-01069 Dresden, Germany*
[2]*Leibniz-Institut für Festkörper- und Werkstoffforschung (IFW) Dresden, D-01171 Dresden, Germany*
[3]*Laboratory for Muon Spin Spectroscopy, Paul Scherrer Institut, CH-5232 Villigen PSI, Switzerland*
[4]*Institute for Theoretical Physics, ETH Zurich, CH-8093 Zurich, Switzerland*
[5]*National Institute for Materials Science, Tsukuba 305-0003, Japan*
[6]*Department of Physics, Kyoto University, Kyoto 606-8502, Japan*
[7]*Max Planck Institute for Chemical Physics of Solids, D-01187 Dresden, Germany*
[8]*School of Physics and Astronomy, University of Birmingham, Birmingham B15 2TT, United Kingdom*

Supplementary Note 1:
Specific heat of $Sr_{2-y}La_yRuO_4$ at ambient pressure

The specific heat measurements were performed at ambient pressure for several pieces of $Sr_{2-y}La_yRuO_4$ single crystals.

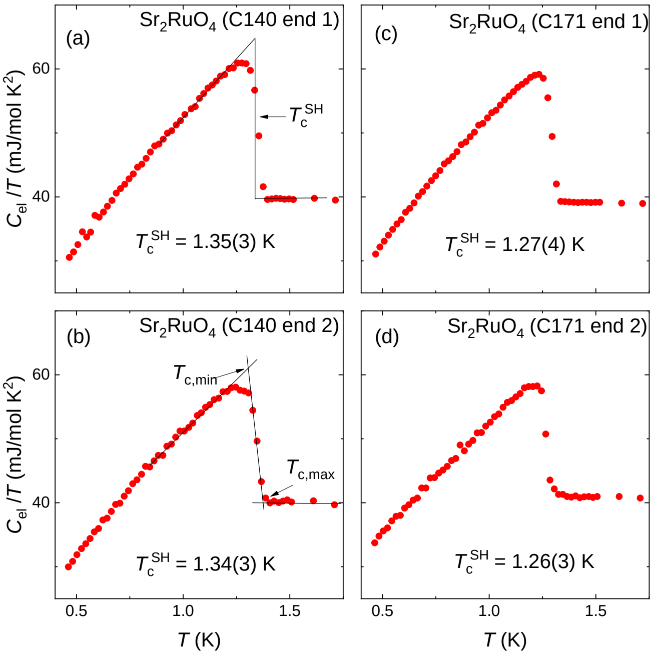

Supplementary Figure 1: Specific heat curves taken for four ending pieces of C140 and C171 Sr_2RuO_4 rods. The mean value of the 'specific-heat' superconducting transition temperature T_c^{SH} is obtained by an equal-entropy construction of the idealized specific heat jump [panel (a)]. The minimum and maximum values of the transition temperature ($T_{c,min}$ and $T_{c,max}$) are determined from the crossing points of linearly extrapolated C_{el}/T vs. T curves in the vicinity of T_c [panel (b)].

For Sr_2RuO_4 used in hydrostatic pressure measurements, the electronic specific heat capacity C_{el}/T was measured for four samples: one sample cut from each end of both the C140 and C171 sections. Results are presented in Fig. 1. The specific-heat critical temperature T_c of each sample was obtained by an equal-entropy construction, illustrated in panel (a). The spread on the critical temperature of each sample is taken as $T_{c,\,max} - T_{c,\,min}$, where $T_{c,\,max}$ and $T_{c,\,min}$ are determined for each transition as illustrated in panel (b). T_c was found to be 1.35(3) and 1.34(3) K for the two samples from rod C140, and 1.27(4) and 1.26(3) for those from C171. Because both rods were used in the hydrostatic pressure measurements, we take a combined value $T_c^{SH} = 1.30(6)$ K for the specific-heat critical temperature of these samples together.

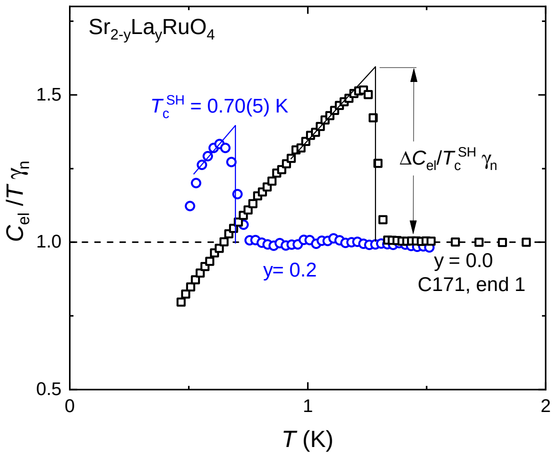

Supplementary Figure 2: Temperature dependence of the normalized electronic specific heat $C_{el}/T\gamma_n$ measured on a small piece cut from the $Sr_{1.98}La_{0.02}RuO_4$ μSR sample (blue open circles). Open squares correspond to the $C_{el}/T\gamma_n$ vs. T data for one of Sr_2RuO_4 samples [C171, end 1; Fig. 1(c)]. Solid lines represent an equal-entropy construction used to determine the superconducting transition temperature T_c. The double-sided arrow represent the way of determination of the specific heat jump at T_c ($\Delta C_{el}/T_c\gamma_n$).

The temperature dependence of C_{el}/T for a small piece cut from the $Sr_{1.98}La_{0.02}RuO_4$ μSR sample is presented in Fig. 2. The C_{el}/T data are normalised by the Sommerfeld coefficient γ_n. The equal-entropy construction and estimates of $T_{c,min}$ and $T_{c,max}$ result in $T_c^{SH} = 0.70(5)$ K.

Note that the 'normalised' data representation allows to make a direct comparison of the specific heat jump

at T_c ($\Delta C_{el}/T_c\gamma_n$) for samples with different La-doping level, as they measured in the present study, as well as for strained Sr_2RuO_4 samples from Ref. 1.

Supplementary Note 2:
ZF- and TF-μSR results at $p = 0.25$ and $p = 0.62$ GPa

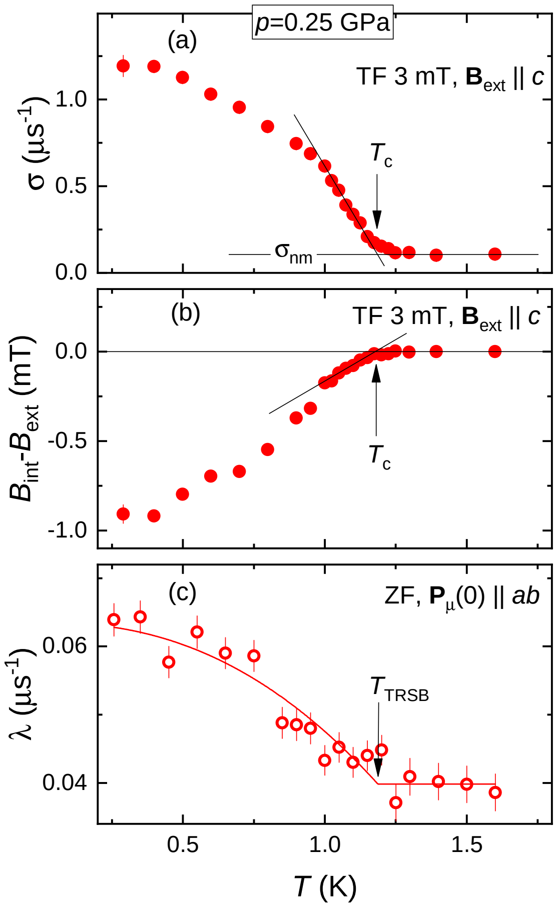

Supplementary Figure 3: (a) Temperature dependence of the Gaussian relaxation rate σ measured at $p = 0.25$ GPa and the external field $B_{ext} = 3$ mT applied parallel to the crystallographic c-axis. (b) The diamagnetic shift of the internal field $B_{int} - B_{ext} \propto M_{FC}$ [2], M_{FC} is the field-cooled magnetization, at $p = 0.25$ GPa. Arrows in panels (a) and (b) indicate the position of the superconducting transition temperature T_c. (c) Temperature dependence of the ZF exponential relaxation rate λ induced by spontaneous magnetic fields caused by TRSB effects at $p = 0.25$ GPa. The initial muon spin polarization $\mathbf{P}_\mu(0)$ is parallel to the ab-plane. The solid line is the fit by means of Eq. 1 from the main text. Arrow indicate the position of TRSB transition temperature T_{TRSB}.

Figures 3 and 4 show the results of TF- and ZF-μSR measurements on Sr_2RuO_4 at $p = 0.25$ and 0.62 GPa. Arrows in panels (a) and (b) indicate the position of the superconducting transition temperature T_c. Arrow in panel (c) indicate the TRSB transition temperature T_{TRSB}.

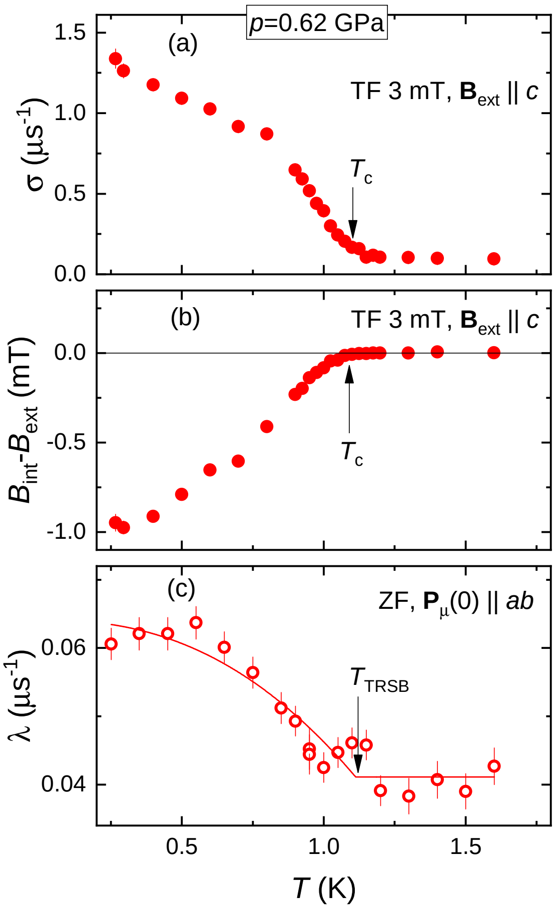

Supplementary Figure 4: The same as in Fig. 3 but for $p = 0.62$ GPa.

Supplementary Note 3:
Extraction of T_c from the TF-μSR data

The superconducting transition temperature T_c was extracted from temperature dependencies of the Gaussian relaxation rate, σ, and the diamagnetic shift of the internal field, $B_{int} - B_{ext} \propto M_{FC}$, as they obtained in TF-μSR experiments [see Figs. 2(b,c), 3(a,b) in the main text and Figs. 4(a,b)].

In a case of $\sigma(T)$ data, the transition temperature was defined as a crossing point of linearly extrapolated $\sigma(T)$ curve in the vicinity of T_c with $\sigma = \sigma_{nm}$ line. Note that σ_{nm} is constant over the entire temperature range and it corresponds to the value reached above T_c [see Fig. 3(a)].

From the diamagnetic shift data, the transition temperature was defined as a crossing point of linearly extrapolated $B_{int} - B_{ext}$ vs. T curve in the vicinity of T_c with $B_{int} - B_{ext} = 0$ line [see Fig. 3(b)].

---

* Electronic address: v.grinenko@ifw-dresden.de
† Electronic address: mansigri@ethz.ch
‡ Electronic address: rustem.khasanov@psi.ch

[1] Li, Y.-S., Kikugawa, N., Sokolov, D. A., Jerzembeck, F.,

Gibbs, A. S., Maeno, Y., Hicks, C. W., Nicklas, M. & Mackenzie, A. P. High sensitivity heat capacity measurements on Sr_2RuO_4 under uniaxial pressure. *PNAS* **118** e2020492118; https://doi.org/10.1073/pnas.2020492118

[2] Weber, M., Amato, A., Gygax, F. N., Schenck, A., Maletta, H., Duginov, V. N., Grebinnik, V. G., Lazarev, A. B., Olshevsky, V. G., Pomjakushin, V. Yu., Shilov, S. N., Zhukov, V. A., Kirillov, B. F., Pirogov, A. V., Ponomarev, A. N., Storchak, V. G., Kapusta, S. & Bock, J. Magnetic-flux distribution and the magnetic penetration depth in superconducting polycrystalline $Bi_2Sr_2Ca_{1-x}Y_xCu_2O_{8+\delta}$ and $Bi_{2-x}Pb_xSr_2CaCu_2O_{8+\delta}$. *Phys. Rev. B* **48**, 13022 (1993).
